# Fear of COVID-19 and hygiene behaviors: a study of secondary school students in İstanbul

**DOI:** 10.55730/1300-0144.6087

**Published:** 2025-10-06

**Authors:** Zozan GÖKSU, Hayriye KIRKOYUN UYSAL, Mehmet DEMİRCİ, Dilek DÜLGER, Seda EKİCİ

**Affiliations:** 1 Department of Public Health, Institute of Health Sciences, Kırklareli University, Kırklareli, Turkiye; 2 Department of Medical Microbiology, Faculty of Medicine, İstanbul University, İstanbul, Turkiye; 3 Department of Medical Microbiology, Faculty of Medicine, Kırklareli University, Kırklareli, Turkiye; 4 Department of Medical Microbiology, Ankara City Health Application and Research Center, University of Health Sciences, Ankara, Turkiye; 5 Veterinary Control Central Research Institute, Ankara, Turkiye

**Keywords:** COVID-19, hand hygiene, personal hygiene, FCV-19S score, student

## Abstract

**Background/aim:**

This study investigates the relationship between fear of COVID-19, measured using the Fear of COVID-19 Scale (FCV-19S), and the hygiene behaviors of secondary school students, assessed with the Hygiene Behavior Scale (HBS).

**Materials and Methods:**

Included in this cross-sectional study were 206 secondary school students. The study data were analyzed using multivariate linear regression.

**Results:**

No relationship was found between the fear of COVID-19 scale (FCV-19S) score of the student sample and the mean total HBS and subdimension values (p > 0.05). It was noted that positive hygiene behaviors decreased as the scale score increased. Students with retired mothers scored 4.463 units higher in the personal hygiene subdimension than those whose mothers were housewives, while the students whose mothers were in blue-collar roles scored 4.868 units higher on the personal hygiene subdimension than those whose mothers were housewives (p < 0.05). The students whose mothers were retired scored 3.845 units higher on the hand-washing subdimension than those whose mothers were housewives (p < 0.05), while those with retired mothers scored 4.052 units higher on the food hygiene subdimension than those whose mothers were housewives (p < 0.05). Students whose mothers were retired scored 12.361 units higher on the HBS than those whose mothers were housewives, while those whose mothers were blue-collar workers scored 7.884 units higher on the HBS than those whose mothers were housewives (p < 0.05). The total mean HBS score of the sample was 48.25 ± 14.41, while the total mean FCV-19S score was 15.18 ± 7.69.

**Conclusion:**

No significant relationship was noted between the Fear of COVID-19 Scale (FCV-19S) scores and hygiene behaviors of secondary school students. Significantly higher total and subdimension HBS scores were noted in students whose mothers were retired or employed than in those whose mothers were housewives. These findings suggest that maternal employment status may play an influential role in the hygiene behaviors of students.

## 1. Introduction

The Coronavirus Disease 2019 (COVID-19) outbreak, caused by SARS-CoV-2 that emerged in 2019, rapidly developed into a global public health crisis. COVID-19 presents with a variety of symptoms, ranging from mild respiratory problems to severe pneumonia, and further to acute respiratory distress syndrome (ARDS).*

It was found that transmission occurs primarily through respiratory droplets, and a number of preventive measures were prescribed, including wearing masks, social distancing, and vaccinations, to control the spread of the disease.†

Aside from the immediate health impacts of COVID-19, there were also profound socioeconomic consequences. The imposed quarantines, travel restrictions, and disruptions to daily life pushed global healthcare systems and economies to their limits [1].⁑

The main strategies to tackle the pandemic sought to curb the spread of the disease into more geographies, but they had a detrimental effect on both mental and physical health. In short, the methods adopted to manage the spread of the disease and to protect physical health had implications for mental health. Restrictions such as home isolation, avoiding crowded environments, social distancing, and limiting contact with those who are high-risk are contrary to human nature, with the potential to lead to serious mental health problems [2]. The common psychological symptoms observed in those who are isolated or quarantined included fear, anxiety, stress, panic attacks, irritability, restlessness, alertness, feelings of helplessness, health anxiety, muscle pain, guilt, worthlessness, reluctance, concentration issues, loss of motivation, increased appetite, feelings of irritability, intolerance, insomnia, feelings of loneliness, and exhaustion [3].

It has been reported that people have different psychological reactions to new pandemics, with the most commonly reported psychological symptoms being fear and anxiety. Health anxiety has been identified as a beneficial function in response to physical discomfort at certain anxiety levels. This normal level of anxiety can ensure the necessary precautions are taken to alleviate risks and prevent illness. When anxiety becomes chronic and excessive, however, health concerns become a problem [4].

Personal hygiene plays an important role in preventive healthcare, in which the aim is to prevent disease before it occurs [5]. Inadequate hygiene has been identified as a leading cause of illness in school-age children and contributes to the spread of infectious diseases in this age group [6]. Personal hygiene refers to the personal care measures people adopt as part of a healthy lifestyle, which contribute to feelings of comfort and confidence. Individual and cultural differences and socioeconomic conditions have all been found to affect hygiene. Personal hygiene is essential, as incorrect or inadequate hygiene can be detrimental to health [7].

Serving as a bridge between the primary and higher education levels, secondary schools seek to support the cognitive and affective development of students [6]. Most healthcare habits are acquired during childhood and adolescence. Failure to take timely precautions to counter health problems that occur during childhood and/or adolescence can pave the way for the emergence of health problems in later years. Furthermore, young people who lack a basic awareness of hygiene may raise unhealthy offspring with similar issues in the future, continuing the vicious cycle. In the longer term, young people are the workforce of the future, and any health problems they carry and perpetuate can be detrimental to social and economic development [8].

On the other hand, excessive hygiene, seen more frequently in developed countries, has also been linked to various health problems. Concerns have been raised over the extensive use of antibacterial soaps, disinfectants, and other hygiene products and their possible adverse effects on health. The obsessive pursuit of cleanliness can disrupt the natural microbial balance in the skin and the environment, with undesirable effects on the immune system [9,10]. The COVID-19 pandemic has had a significant effect on the hygiene habits of all age groups, including students. The present study investigates the relationship between fear of COVID-19 and the hygiene practices of secondary school students to contribute to the raising of healthy individuals in the future. Specifically, the present study investigates the relationship between fear of COVID-19 and hygiene behaviors among the students of a secondary school in İstanbul.

## 2. Materials and methods

### 2.1. Study design

This cross-sectional descriptive study was conducted in a private secondary school in İstanbul between March and June 2023. The tools used for the collection of data from the students were a literature-based Introductory Characteristics Information Form, the Hygiene Behaviors Scale, and the COVID-19 Fear Scale. WHO declared the COVID-19 global emergency over on May 23, 2023. The present study was conducted between March and June 2023, in the final phase of the COVID-19 pandemic, and sought to identify whether residual fear of the pandemic was continuing to influence the hygiene behaviors of students after restrictions had been lifted.

### 2.2. Data collection

Prior to the study, ethics committee approval was obtained from the Kırklareli University Health Sciences Institute Ethics Committee (Date: 01.03.2023, Number: E-69456409-199-79846). Furthermore, permission for the use of the scales was obtained from their respective authors, while institutional permission was received from the secondary school involved in the study. The purpose of the study was explained to the students and their parents, who subsequently provided written informed consent for their inclusion.

The age, sex, grade, number of siblings, family type, education level, professions of the mother and father, and economic status were recorded on the Introductory Characteristics Information Form. The Hygiene Behavior Scale (HBS) used in the study, developed by Çoban and Bilgin in 2015 [11], contains no medical terms, and can be used to measure the hygiene behaviors of the respondent in a social context. The scale contains 25 questions spanning three subdimensions of hygiene: personal, hand washing, and food hygiene, and answers are given on a 4-point (1–4) Likert-type scale. The first 13 items of the scale evaluate the personal hygiene subdimension, items 14–19 evaluate the hand washing subdimension, and items 20–25 evaluate the food hygiene subdimension. The higher the total score from the scale, the lower the positive hygiene behavior. The possible range of scores is 25–100, with scores of 38 and above being indicative of negative hygiene behaviors. The minimum and maximum mean scores in the personal hygiene subdimension were 13 and 52, respectively, and those who scored 20 points and above in this subdimension were considered to have negative hygiene behaviors. For the hand washing and food hygiene subdimensions, the possible minimum and maximum scores were 6 and 24, with scores of 9 points and above indicating negative hygiene behavior. The adaptation of the Fear of COVID-19 Scale (FCV-19S: The Fear of COVID-19 Scale) to the Turkish context was conducted by Ahorsu et al. in 2020 [12], and its validity and reliability analysis was carried out by Satıcı et al. in 2021 [13]. The scale is considered suitable for a wide age range, and especially for university students and adults. The scale comprises seven items, subject to a positive scoring system. Responses are given on a 5-point Likert-type scale, and there are no reverse-scored items. Possible total scores are in the range of 7–35, with higher scores indicating a high level of fear associated with the COVID pandemic. No cut-off point is set.

### 2.3. Statistical analysis

For the analysis of the data acquired from the secondary school students, frequency distributions were used for categorical characteristics while descriptive statistics were applied to numerical values. The normality of distribution was tested with a Shapiro-Wilk test, while an independent sample t-test and one-way ANOVA were used to investigate the differences between the scale scores and the categories of the variables investigated. Relationships between numerical characteristics were assessed using the Pearson correlation coefficient, linear regression was used to determine the variables affecting hygiene behaviors, and p < 0.05 was set as the significance level.

## 3. Results

The mean age of the students was 12.13 ± 1.13, 47.6% were female, 44.7% were in the 8th grade, 84% had siblings, and the mean value number of siblings was 2.13 (min-max:1–6). Furthermore, 71.8% (n = 148) of the students had a nuclear family structure, 37.9% of the mothers had a university education level or above, 41.2%, and 35% had a high school education father and mother respectively. It was found that the 6.8% of mother’s profession was retired, 56.7% of the fathers were self-employed, and 55.8% of them had a moderate economic level (Table 1).

The mean total HBS score, and the mean Personal hygiene, Hand washing, and Food hygiene subdimension scores were 48.25 ± 14.41, 25.98 ± 7.67, 11.40 ± 4.23, and 10.87 ± 4.03, respectively. The mean FCV-19S score was 15.18 ± 7.69 (Table 2). Cronbach’s alpha values were calculated for the reliability of the scales and were above 0.70, confirming the reliability of the applied scales (Table 2).

The HBS and FCV-19S scores were found to be unaffected by sex, class, presence of siblings, family type, father’s education level, and economic status of the family (p > 0.05) (Table 3).

The hand-washing subdimension score was found to be significantly affected by the educational status of the mother (p < 0.05), being higher among the students whose mothers were primary school graduates than in those whose mothers were high school graduates.

Statistically significant differences were noted between the effects of maternal professions on HBS and FCV-19S scores (p < 0.05). Personal hygiene subdimension scores were higher among the children of mothers who were retired or in blue-collar professions than in those of mothers in other professions, while the total HBS and hand washing and food hygiene subdimension scores were higher among those whose mothers were retired than those whose mothers were housewives, civil servants or self-employed. Furthermore, FCV-19S scores were lower among those whose mothers were blue-collar workers than those with mothers in other professions (Table 3).

Significant differences were noted in the effects of the fathers’ professions on the personal hygiene and hand washing subdimensions, overall hygiene behavior and FCV-19S scores (p < 0.05). The total HBS scores, and personal hygiene and hand washing subdimension scores were higher among the students with fathers who were retired or in blue-collar jobs than in those whose fathers were self-employed. Furthermore, FCV-19S scores were higher in those whose fathers were retired than in those whose fathers were blue-collar workers or self-employed (Table 3). The age and number of siblings had no significant effect on HBS and FCV-19S scores (p > 0.05) (Table 4), and no significant relationship was noted between the HBS and COVID-19 Fear Scale scores (p > 0.05) (Table 5).

Maternal profession was found to have a significant impact on the total HBS and subdimension scores. The personal hygiene subdimension score of students whose mothers were retired was 4.463 units higher than that of those whose mothers were housewives, while the personal hygiene subdimension score of those whose mothers were blue-collar workers was 4.868 units higher than that of those whose mothers were housewives. The hand-washing subdimension score of the students whose mothers were retired was 3.845 units higher than that of those whose mothers were housewives. The food hygiene subdimension score of the students whose mothers were retired was 4.052 units higher than that of those whose mothers were housewives. The HBS score of the students whose mothers were retired was 12.361 units higher than that of those whose mothers were housewives, and the HBS score of those whose mothers were blue-collar workers was 7.884 units higher than that of those whose mothers were housewives (Table 6).

## 4. Discussion

Understanding the relationship between FCV-19S scores and the hygiene behaviors of secondary school students was crucial for the development of targeted public health interventions during the pandemic [12,14]. The effects of fear on mental health included anxiety and stress, leading potentially to preventive behaviors, but also irrational practices or misinformation [15–17]. A comprehensive examination of this relationship could thus be considered necessary for the design of interventions that could promote adaptive behaviors. To this end, the present study was conducted to investigate the FCV-19S scores and hygiene behaviors of secondary school students in İstanbul.

According to the results of the present study, the fear of COVID-19 did not influence the mean scores on the HBS or its subdimensions among secondary school students. On the other hand, it was noted that students whose mothers were retired or working scored higher in the personal hygiene, hand washing, and food hygiene subdimensions of the HBS, and in the overall HBS than students whose mothers were housewives. Although FCV-19S scores did not influence hygiene behaviors in the present study, psychosocial support services, stress management resources, peer support programs, and parental guidance are all recommended to ensure hygiene behaviors are maintained at optimal levels without being driven by excessive fear. Despite being conducted in a single school and after the peak pandemic period, the present study provides valuable insights into the impact of fear of COVID-19 among children on their hygiene behaviors.

FCV-19S scores were found to have no effect on either the total HBS score or the mean subdimension values. Contrasting the findings of the present study, a nationwide study of secondary school students in Poland evaluating hand hygiene behaviors during the COVID-19 pandemic and investigating the impact of the COVID-19 pandemic on these behaviors found hygiene behaviors to be significantly improved after the COVID-19 pandemic [18]. In a study by Lee et al. investigating the determinants of behavioral changes in secondary school students in South Korea since COVID-19, it was revealed that the increased perception of risk spurred by COVID-19 had led to more protective behavior changes among secondary school students [19]. In a study conducted in Türkiye to identify the factors affecting the fear levels of secondary school students during the COVID-19 pandemic, it was found that students took such precautions as home isolation, wearing masks, paying attention to hygiene, and social distancing to reduce their fear levels during the COVID-19 pandemic [20]. In further studies, it was reported that those experiencing high fear levels were more likely to resort to such preventive measures as frequent hand washing, wearing masks, and social distancing [15,16]. The relationship between fear and hygiene behaviors is complex, and the psychological impacts of fear can lead to increases in hygiene behaviors. Although fear encourages positive behaviors, it might also lead to irrational or excessive hygiene practices. Overemphasizing certain behaviors may promote unnecessary anxiety or the spread of misinformation. Understanding the relationship between fear and hygiene is crucial to avoid the negative consequences that excessive fear might cause [17]. No relationship was identified between fear of COVID-19 and hygiene behaviors in the present study, which may be attributable to the fact that the pandemic was nearing its end at the time of the study, and so fear of the disease was declining.

In a study of nurses, a positive relationship was reported between FCV-19S score and hygiene behaviors [21]. In a study conducted by Sari and Bilmez investigating the impact of COVID-19 on oral hygiene in a sample of adults, it was found that adults with a high FCV-19S score had started to pay closer attention to oral hygiene and to use more oral care products [22]. The positive relationship between FCV-19S score and hygiene behaviors identified in previous studies has been attributed to various factors. Fear of COVID-19 can lead to anxiety and stress, especially among vulnerable middle school students. Studies have shown that excessive fear and anxiety can affect mental health with an effect on both cognitive processes and decision-making [12,14].

Wang et al. investigated the impact of COVID-19 fear on mental health among 11,872 college students in a metaanalysis of data from 16 studies, quantifying their fear using the FCV-19S. An average FCV-19S score of 17.60 was calculated, indicating moderate fear, and women were noted to experience higher levels of fear compared to men. The highest fear scores were identified in the studies conducted in Israel, Russia and Belarus, while the lowest scores were reported in Europe. The authors concluded that COVID-19 had had a significant effect on the mental health of students, highlighting the need for targeted prevention programs [23]. Lathabhavan explored how fear of COVID-19 had affected the well-being and life satisfaction of college students in India through a survey of 768 students during the first wave and 884 students during the second wave of the COVID-19 pandemic. The study revealed a link between increased psychological distress and decreased well-being and life satisfaction, and reported a more pronounced impact during the second wave. Psychological distress was thus found to mediate the relationship between COVID-19 fear and both well-being and life satisfaction [24]. Doğanülkü et al. investigated how intolerance of uncertainty mediated the relationship between fear of COVID-19 and procrastination among 450 Turkish university students in a study conducted between October and November 2020. The study identified a positive link between fear of COVID-19 and both intolerance of uncertainty and procrastination [25]. The difference between the results of this study and those of the present study can be attributed to the fact that our study was conducted after the peak epidemic period, and the younger age of the student cohort.

Maternal occupation was noted to have a significant impact on the overall HBS and subdimension scores. The personal hygiene subdimension scores of the students with retired mothers was 4.463 units higher than that of those whose mothers were housewives, while the personal hygiene subdimension score of those whose mothers were blue-collar workers was 4.868 units higher than that of those whose mothers were housewives. The hand-washing subdimension score of the students with retired mothers was 3.845 units higher than that of those whose mothers were housewives; while the food hygiene subdimension score of the students with retired mothers was 4.052 units higher than that of those whose mothers were housewives. The HBS score of the students with retired mothers was 12.361 units higher than that of those with mothers who were housewives, while the HBS score of those whose mothers were workers was 7.884 units higher than that of those whose mothers were housewives. Similar to the results of the present study, in a study conducted in Türkiye by Ayran et al. investigating the factors affecting the hand hygiene and mask-wearing behaviors of secondary school students during the COVID-19 pandemic, maternal profession was found to have an effect on hand hygiene and mask-wearing behaviors [26]. The study reported better hand hygiene and mask-wearing behaviors among students with employed mothers when compared to those whose mothers were not gainfully employed [26]. In a study investigating the factors affecting hand hygiene and mask-wearing among primary school students in Wuhan, China, it was found that the profession of the mother had a significant effect on the individual hand hygiene and mask-wearing behaviors of students [27]. The study further reported that lower hand hygiene and mask-wearing behaviors among children whose mothers were not gainfully employed or employed in temporary positions. It was noted, however, that when other familial and individual characteristics were also taken into account, the mother’s occupation lost its significance in terms of its impact on the hand hygiene and mask-wearing behaviors of students [27]. The results of the present study reveal that, on the whole, the hand, food, and personal hygiene practices of students whose mothers were in gainful employment were poorer than those whose mothers were housewives. The better hygiene behaviors of children whose mothers are housewives may be attributable to the greater time mothers who are housewives can spend taking care of their children. The results of previous studies have offered other explanations. Mothers in gainful employment have set daily routines for food, hand washing, and personal hygiene, and such routines may have a positive impact on children by contributing to their development of better hygiene practices. Children tend to copy the behaviors of their parents, and working mothers may promote effective hygiene habits in their children by setting an example and encouraging their children. Women working in professional settings can access the most up-to-date information and resources related to hygiene, which may translate into more informed decisions about hand washing, food safety, and personal hygiene for those who are mothers. The children of mothers in gainful employment may develop self-confidence as a result of their increased responsibility and independence in matters related to personal hygiene. When parents are busy with work-related tasks, children may learn to manage their hygiene routines with less direct supervision. Furthermore, children in families with working mothers may develop stronger adaptation and resilience skills, and these may extend into the maintenance of good hygiene practices during such challenging times such as pandemics. The ability of children to adapt to changing conditions may support them in being more proactive when it comes to hygiene. In addition, mothers who work may be better placed to communicate openly with their children about the importance of hygiene, which can be especially important during outbreaks, leading to a shared understanding and commitment to the maintenance of good hygiene practices. Mothers in gainful employment may also have the necessary financial resources for the purchase of hygiene products and the creation of a healthy home environment, contributing to the overall hygiene of the household. It should not be assumed, however, that the children of working mothers will develop better hygiene habits, as cultural norms, socioeconomic status, and the overall family environment can also be influential. Every family is unique, and individual circumstances can vary considerably.

## 5. Conclusion

No significant relationship between the fear of COVID-19, measured using the FCV-19S, and the hygiene behaviors of secondary school students was identified in the present study. That said, a notable link was identified between maternal employment and hygiene practices, with the students whose mothers were retired or employed recording higher scores in personal hygiene, hand-washing, food hygiene, and overall hygiene behaviors than those whose mothers were housewives. These findings suggest that among the parental factors, maternal employment in particular plays an important role in shaping the hygiene habits of students. Although the study was conducted in a single school and after the pandemic had reached its peak, it highlights the importance psychosocial support, stress management, peer support programs, and parental guidance for the maintenance of optimal hygiene behaviors in children without being driven by excessive fear.

## Figures and Tables

**Table 1:** Sociodemographic characteristics of participants.

		n	%
Age mean ± SD (min–max)		12.80 ± 1.09 (11–15)	
Sex	Male	108	52.4
	Female	98	47.6
Grade	5th grade	21	10.2
	6th grade	33	16.0
	7th grade	60	29.1
	8th grade	92	44.7
Sibling	Yes	173	84.0
	None	33	16.0
Number of siblings mean ± SD (min–max)		2.13 ± 1.13 (1–6)	
Family type	Nuclear family	148	71.8
	Extended family	45	21.8
	Single-parent family	13	6.4
Mother’s education level	Illiterate	8	3.9
	Primary school	22	10.7
	Middle school	26	12.6
	High school	72	35.0
	University and above	78	37.8
Father’s education level	Illiterate	5	2.4
	Primary school	22	10.7
	Middle school	22	10.7
	High school	85	41.2
	University and above	72	35.0
Mother’s profession	Housewife	101	49.0
	Retired	14	6.8
	Employee	21	10.2
	Office worker	27	13.1
	Self-employed	43	20.9
Father’s profession	Retired	10	4.9
	Employee	56	27.2
	Office worker	23	11.2
	Self-employed	117	56.7
Economic status of the family	Good	82	39.8
	Moderate	115	55.8
	Poor	9	4.4

**Table 2:** Descriptive statistics on scale and subdimensions.

	Mean	SD	Min	Max	Cronbach’s alpha
Hygiene Behaviors Scale	48.25	14.41	25	98	0.910
Personal hygiene subdimension	25.98	7.67	13	52	0.828
Hand washing subdimension	11.40	4.23	6	24	0.777
Food hygiene subdimension	10.87	4.03	6	24	0.756
FCV-19S score	15.18	7.69	5	35	0.916

**Table 3:** Relationship between Hygiene Behaviors Scale and subdimension scores and sociodemographic characteristics.

	Personal hygiene	Hand-washing	Food hygiene	Hygiene Behaviors Scale	FCV-19S score
	Mean±SD	Mean±SD	Mean±SD	Mean±SD	Mean±SD
**Sex**					
Male	26.97±8.17	11.77±4.34	11.18±4.18	49.92±15.14	15.00±8.18
Female	24.88±6.95	11.00±4.10	10.53±3.85	46.41±13.40	15.38±7.14
**t/p**	1.971/0.050	1.303/0.194	1.149/0.252	1.754/0.081	−0.351/0.726
**Grades**					
5	26.52±10.35	11.62±5.13	11.33±4.92	49.48±18.75	19.05±8.95
6	24.79±6.90	10.64±3.97	9.76±3.32	45.18±12.48	15.61±6.24
7	25.52±6.71	11.60±3.94	10.87±3.67	47.98±13.18	14.22±7.07
8	26.58±7.87	11.50±4.33	11.16±4.25	49.24±14.77	14.77±8.07
**f/p**	0.555/0.645	0.435/0.728	1.094/0.353	0.698/0.554	2.247/0.084
**Siblings**					
Yes	25.86±7.51	11.44±4.18	10.92±4.07	48.23±14.16	15.29±7.79
None	26.58±8.58	11.21±4.57	10.58±3.86	48.36±15.90	14.58±7.21
**t/p**	−0.489/0.625	0.282/0.778	0.455/0.649	−0.050/0.960	0.492/0.624
**Family type**					
Nuclear family	25.71±7.13	11.29±4.04	10.79±3.79	47.79±13.23	14.65±7.40
Extended family	26.93±9.37	11.80±4.89	11.16±4.83	49.89±18.08	17.07±8.46
**t/p**	−0.947/0.345	−0.711/0.478	−0.470/0.640	−0.727/0.470	−1.874/0.062
**Mother’s education level**					
Primary school	27.38±8.49	12.59±4.84a	11.45±4.23	51.41±15.96	16.98±8.40
High school	25.36±6.38	10.69±3.55b	10.53±3.42	46.58±12.10	14.85±7.12
University and above	25.54±8.10	11.21±4.22	10.77±4.39	47.51±15.01	14.19±7.53
**f/p**	1.293/0.277	**3.367/0.036** *	0.856/0.427	1.949/0.145	2.279/0.105
**Father’s education level**					
Primary school	27.98±8.21	12.49±4.91	11.51±4.45	51.98±16.24	14.84±7.77
High school	25.27±6.53	11.00±3.74	10.55±3.66	46.82±12.33	15.25±7.43
University and above	25.44±8.37	11.14±4.23	10.81±4.15	47.39±15.12	15.33±8.02
**f/p**	2.231/0.110	2.163/0.118	0.890/0.412	2.212/0.112	0.066/0.936
**Mother’s profession**					
Housewife	25.47±6.82b	10.94±3.75b	10.38±3.41b	46.78±12.09b	14.74±6.98a
Retired	29.93±10.28a	14.79±5.87a	14.43±5.65a	59.14±20.71a	17.00±11.01a
Employee	30.33±10.14a	12.38±5.08	11.95±4.89	54.67±19.15	11.14±5.47b
Office worker	24.26±6.98b	10.85±3.87b	10.41±3.76b	45.52±13.32b	17.33±7.67a
Self-employed	24.84±6.79b	11.26±4.08b	10.63±4.00b	46.72±13.26b	16.23±8.39a
**f/p**	**3.470/0.009** *	**3.061/0.018** *	**3.813/0.005** *	**3.872/0.005** *	**2.531/0.042** *
**Father’s profession**					
Retired	29.10±10.48a	13.50±5.46a	12.30±5.36	54.90±20.68a	21.20±10.41a
Employee	28.48±8.50a	12.36±4.59a	11.46±4.33	52.30±16.09a	14.50±7.90b
Office worker	26.30±9.11	11.52±4.21	10.83±4.01	48.65±15.87	17.48±8.52
Self-employed	24.44±6.26b	10.74±3.84b	10.47±3.75	45.66±12.03b	14.54±6.91b
**f/p**	**4.314/0.006** *	**2.788/0.042** *	1.214/0.306	**3.586/0.015** *	**3.251/0.023** *
**Economic status of the family**					
Good	26.09±9.05	11.20±4.45	11.10±4.56	48.38±16.46	14.20±7.77
Moderate	25.90±6.64	11.54±4.10	10.72±3.65	48.16±12.95	15.83±7.59
**t/p**	0.156/0.876	−0.572/0.568	0.632/0.528	0.100/0.920	−1.499/0.135

*a, b: Indicates mean differences between groups (a: highest mean)*.

*F: One-way ANOVA, t: Independent sample t-test*,

* *: p < 0.05*.

**Table 4:** Relationship between age and number of siblings and Hygiene Behaviors Scale and subdimensions.

		Personal hygiene	Hand-washing	Food hygiene	Hygiene behaviors scale	FCV-19S score
Age	r	0.103	0.100	0.122	0.118	−0.056
	p	0.140	0.151	0.082	0.090	0.423
Number of siblings	r	−0.075	−0.014	−0.022	−0.050	0.114
	p	0.326	0.852	0.775	0.511	0.137

*r: Pearson correlation coefficient*,

* *: p < 0.05*.

**Table 5:** Relationship between scales.

		Personal hygiene	Hand-washing	Food hygiene	Hygiene behaviors scale	FCV-19S score
Personal hygiene	r	1	0.704	0.688	0.932	−0.038
	p		**0.000** *	**0.000** *	**0.000** *	0.590
Hand-washing	r		1	0.773	0.885	−0.067
	p			**0.000** *	**0.000** *	0.336
Food hygiene	r			1	0.873	0.004
	p				**0.000** *	0.959
Hygiene behaviors scale	r				1	−0.039
	p					0.579
FCV-19S score	r					1
	p					

*r: Pearson correlation coefficient*,

* *: p < 0.05*.

**Table 6:** Factors affecting HBS and subdimension scores. p-values shown in bold represent statistically significant results.

	Unstandardized coefficient		Standardized coefficient	t	p	95.0% CI	
	B	Std. Error	Beta			Lower limit	Upper limit
**Personal hygiene subdimension**							
(Constant)	25.465	0.745		34.160	0.000	23.995	26.935
Retired/Housewife	4.463	2.137	0.147	2.089	**0.038**	0.250	8.676
Worker/Housewife	4.868	1.797	0.192	2.709	**0.007**	1.325	8.411
Civil servant/housewife	−1.206	1.623	−0.053	−0.743	0.458	−4.407	1.994
Self-employed/Housewife	−0.628	1.364	−0.033	−0.460	0.646	−3.318	2.062
(F: 3.470. p: 0.009. R^2^: 0.065							
**Hand-washing subdimension**							
(Constant)	10.941	0.413		26.484	0.000	10.126	11.755
Retired/housewife	3.845	1.184	0.229	3.248	**0.001**	1.510	6.180
Worker/housewife	1.440	0.996	0.103	1.447	0.150	−0.523	3.404
Civil servant/housewife	−0.089	0.899	−0.007	−0.099	0.922	−1.862	1.685
Self-employed/housewife	0.315	0.756	0.030	0.417	0.677	−1.175	1.806
(F: 3.061. p: 0.018. R ^2^: 0.057							
**Food hygiene subdimension**							
(Constant)	10.376	0.390		26.576	0.000	9.606	11.146
Retired/housewife	4.052	1.119	0.254	3.621	**0.000**	1.846	6.259
Worker/Housewife	1.576	0.941	0.119	1.675	0.096	−0.279	3.432
Civil servant/housewife	0.031	0.850	0.003	0.037	0.971	−1.645	1.707
Self-employed/housewife	0.252	0.714	0.025	0.352	0.725	−1.157	1.661
(F: 3.813. p: 0.005. R^2^: 0.071							
**Hygiene Behaviors Scale**							
(Constant)	46.782	1.395		33.529	0.000	44.031	49.533
Retired/housewife	12.361	3.999	0.216	3.091	**0.002**	4.476	20.246
Worker/housewife	7.884	3.363	0.166	2.344	**0.020**	1.253	14.516
Civil Servant/housewife	−1.264	3.038	−0.030	−0.416	0.678	−7.254	4.727
Self-employed/housewife	−0.061	2.553	−0.002	−0.024	0.981	−5.096	4.973
(F: 3.872. p: 0.005. R^2^: 0.072							

## Footnotes

* World Health Organization (WHO). Coronavirus disease 2019 (COVID-19) situation report – 51. (2020a). [online]. Website https://www.who.int/docs/default-source/coronaviruse/situation-reports/20200311-sitrep-51-COVID-19.pdf?sfvrsn=1ba62e57_10 [accessed 09 June 2024].

† Centers for Disease Control and Prevention [CDC]. COVID-19: How to protect yourself and others. (2021). [online]. Website https://www.cdc.gov/coronavirus/2019-ncov/prevent-getting-sick/prevention.html [accessed 09 June 2024].

⁑ World Bank. Global economic prospects. (2021). [online]. Website https://www.worldbank.org/en/publication/global-economic-prospects [accessed 09 June 2024].
